# Are cyclic plant and animal behaviours driven by gravimetric mechanical forces?

**DOI:** 10.1093/jxb/erab462

**Published:** 2021-11-02

**Authors:** Cristiano de Mello Gallep, Daniel Robert

**Affiliations:** 1 School of Technology, University of Campinas, r. Paschoal Marmo 1888, Limeira/SP, 13484-332, Brazil; 2 School of Biological Sciences, University of Bristol, 24 Tyndall Avenue, Bristol BS8 1TQ, UK; 3 Technical University of Darmstadt, Germany

**Keywords:** Animal activity, biological cycles, circadian biology, gravimetric tide, human activity, plant growth, plant movement

## Abstract

The celestial mechanics of the Sun, Moon, and Earth dominate the variations in gravitational force that all matter, live or inert, experiences on Earth. Expressed as gravimetric tides, these variations are pervasive and have forever been part of the physical ecology with which organisms evolved. Here, we first offer a brief review of previously proposed explanations that gravimetric tides constitute a tangible and potent force shaping the rhythmic activities of organisms. Through meta-analysis, we then interrogate data from three study cases and show the close association between the omnipresent gravimetric tides and cyclic activity. As exemplified by free-running cyclic locomotor activity in isopods, reproductive effort in coral, and modulation of growth in seedlings, biological rhythms coincide with temporal patterns of the local gravimetric tide. These data reveal that, in the presumed absence of rhythmic cues such as light and temperature, local gravimetric tide is sufficient to entrain cyclic behaviour. The present evidence thus questions the phenomenological significance of so-called free-run experiments.

## Teaser

The rhythmic activity of biological organisms is proposed to be closely associated with the gravimetric tides generated by the orbital mechanics of the Sun–Moon–Earth system.

## Introduction

All organisms exhibit cyclical modulations in their levels of activity that are deemed to be of adaptive value. Long-term and short-term cycles are thus ubiquitous and can be regarded as ‘embodied rhythms of life’, a temporally organized homeostatic activity dictated by or even exploiting the cyclic variations of environmental variables. Such variations are diverse and well known; examples are variations in day and night, the passing of the seasons and their associated periods of cold, dark, or wet, or any combination thereof, and also the abundance or lack of resources in ecological niches. Such cyclic variations are ubiquitously found from microorganisms to unicellular and multicellular organisms, including human beings and their socio-economic life, which also crucially depends on natural daily and seasonal rhythms. The 2017 Nobel Prize in Physiology or Medicine was awarded to those who discovered some of the molecular mechanisms underpinning circadian rhythms, providing the first mechanistic insights into how organisms physiologically organize their cyclic activities, in particular to the ~24h period of the Earth’s rotation (Nobel Assembly, 2017).

Biological cycles have long occupied the minds of the keen observers of nature. Early records date back to the early 18th century, with Carl Linnaeus noting that it is possible to predict the closing and opening times of petals for different flowers (Kuhlman *et al.*, 2007). Further observations by the astronomer de Mairan on the persistence of leaf movements even in constant or modified conditions of light and darkness led to the notion that ‘the sensitive plant thus senses the sun without seeing it in any way’ (de Mairan, 1729). Two centuries later, the pioneering chronobiologists Erwin Bünning and Kurt Stern provided the first evidence that a genetic mechanism influenced circadian rhythms by demonstrating its inheritance in bean plants (Bünning and Stern, 1930;Kuhlman *et al.*, 2007). This contribution constituted a seminal building block towards our understanding of a molecular, genetically based, and centrally controlled ‘clock’ working using the interplay of intricate molecular feedback loops, known today as the internal circadian clock (Patke *et al.* 2020).

Since then, great progress has been made in revealing the biological internal mechanisms for sensing environmental cycles and for producing rhythmic processes, be they physiological or behavioural (Kuhlman *et al.*, 2007). Yet, open questions remain, such as whether the functional elements or constitutive components of the internal clock are centralized or distributed—or both—across any given multicellular organism. In other words, can local oscillators exist within single cells, or do they obtain their functionality from larger multicellular interactions? At a larger scale, what is the nature of the interactions between the internal clock, or the distributed clocks, and the external environmental parameters, the so-called *Zeitgeber* (Lüttge, 2003)? What actually constitutes the master oscillator that maintains the periodicities observed in so many organisms and cells at behavioural, physiological, and molecular levels?

While it is trivial to state that any biological activity is not constant with time, the fluctuations observed can vary greatly as a function of the time of day or, for that matter, along shorter or longer time scales. Measurements made on biological systems are therefore very likely to be time sensitive, and so particular attention must be paid to time series unfolding at various scales. The fluctuations that have been long reported to dominate are the well-known circadian cyclic patterns that vary with the succession of day and night (Sweeney, 1977). Hence, natural circadian rhythms have been intimately linked to light/dark cycles and their ubiquity on Earth. Yet, other environmental factors have been proposed and studied in the long history of chronobiology (Aschoff, 1981). A rhythmic sensitivity of biological systems to other, and sometimes very weak, parameters has been studied and demonstrated in many cases (Sweeney, 1977; Brown, 1983; Lüttge, 2003). Notably, a viable hypothesis was expressed in the late 1950s that an internal clock could be also driven by external cyclic cues of a geophysical nature, rather than light/dark cycles only (Brown, 1983). In that view, the clocks were considered to be ‘open systems depending upon subtle geophysical rhythms’ (Brown, 1959).

The chronobiologist Frank A. Brown proposed that interactions with atmospheric electromagnetic forces or other geophysical cyclic processes could act as external drivers of observed biological rhythms, and pointed out that these potential drivers would be ‘always operative and hard to block’. In effect, Brown documented variations in the coloration of crabs organized in cycles of 24h (circadian) and 12.4h and 24.8h (circatidal) (Brown, 1976), and persistent tidal cycles in the activity of oysters in controlled laboratory conditions (Brown, 1954). Notably, these rhythms were observed in experiments run under conditions of constant darkness, for example, the so-called free-running conditions. In oysters, the remarkable feature was the expression of the tidal cycle far away from the seashore, hence remote from actual maritime tides. The persistence of such tidal-like cycles has also been documented in other seashore organisms, including crustacea (Skov *et al.*, 2005; Zhang *et al.*, 2013), annelids, molluscs (Tran *et al.*, 2016), fish (Cresci *et al.*, 2019), and even some insects (Bruce and Pittendrigh, 1957). It was invariably found that these animals modulate their behaviour in tune with the ~12.4h ebb and flow of the water tides. Moreover, when they were moved to a nearby laboratory providing controlled and stable aquatic conditions, this activity cycle was maintained for several days, matching the expected lunisolar tidal timing at the location from where the organisms had been collected in nature (Palmer, 1973; Wilcockson and Zhang, 2008).

Such circatidal cycles of activity also occur in deep-sea invertebrates (Mercier *et al.*, 2011), animals that are not exposed to the actual bulk motion of water tides. Interestingly, for terrestrial mangrove crickets, cyclic circadian locomotor activity appears to depend on the expression of the circadian *Clock* (*Clk*) gene when measured in constant-light conditions, whereas the slightly longer 24.8h circatidal cycle is not affected by interfering with *Clk* expression (Takekata *et al.*, 2014). Persistent circatidal cycles in free-running conditions were found for the stickiness (Straley and Bruce, 1979), the melatonin level (Tal *et al.* 2011), and the gravitactic locomotory behaviour of *Euglena* algae (Lebert, 1999). Rhythmic activity in the breeding of insects was also reported (Bruce and Pittendrigh, 1957), presenting a period of ~24.5h, close to the length of the circatidal day. Similar tide-like cycles are evident in the water distribution inside roots (Takase, 2011), in the respiration of shrimps (Leiva, 2016), in the metabolism of seedlings (Ievinsh and Kreicbergs, 1992), in the growth of moss (Mironov *et al.*, 2020), in bipolar mood cycles and sleep in humans (Cajochen *et al.*, 2013; Wehr, 2018), and in the activity of luminescent fungi (Oliveira *et al.*, 2015), to cite some of the numerous well-documented cases (Lüttge, 2003; Kuhlman *et al.*, 2007).

As circatidal timings emerge as ubiquitous from the literature on biological rhythms (Lüttge, 2003; Kuhlman *et al.*, 2007), a few words seem adequate to introduce the phenomenology of the lunisolar tides. In a first and probably sufficient approximation, the system to consider is that of the Earth, the Moon, and the Sun. From the perspective of any piece of matter on Earth, living or non-living alike, the relative instantaneous positions of Moon and Sun in the sky will determine the magnitude, phase, and direction of the total force of gravity, the lunisolar gravimetric tide and its variations, exerted on that piece of matter. This gravitational force is not only responsible for the tides of oceans, rivers, and wells, but also has substantial effects on the cyclical mechanical oscillations of the Earth’s crust (Arnaudon *et al.*, 1993; Takao and Shimada, 2000; Boerez *et al.*, 2012). As well as impacting man-made structures, the lunisolar tide also affects in a lesser known but no less potent way the cyclic modulations of the Earth’s geomagnetic shield and other geo-electromagnetic phenomena around the world (Akasofu, 1982; Adushkin *et al.*, 2017). Interestingly, the influence of such tidal cycles on organisms has been used to infer the changes in the Earth–Moon system through the ages by quantifying the daily and monthly growth of the Nautilus shell (Kahn and Pompea, 1978).

The gravimetric tide, about a millionth of *g,* is a force of nature that has been pervasive on Earth for as long as days and nights have existed. The question has arisen as to whether local gravimetric tides play any role in biological systems. In effect, the daily, monthly, and annual cycles of the Earth–Sun–Moon orbital system impose a variation of the net gravitational force, called ‘*δg*’ here, on every object with mass. Here, our question pertains to the possible influence of patterns in *δg* on isopods, coral, or a developing plant, and whether *δg* can indeed be regarded as a pervasive and bona fide rhythm generator, or *Zeitgeber.* Could gravity cycles act as an external trigger, an ever-present and very predictably rhythmic environmental parameter that could influence the development and rhythmicity of so-called internal clocks (du Pré *et al.*, 2014)? This question is not new and has been debated for decades, yet with only limited success in resolution (Lüttge, 2003). In addition, would such tidal triggering help us in understanding the Moon-related traditional practices in forestry and agriculture that have been reported and used for so long across the continents (Zürcher, 2001)? Could such a gravitational *Zeitgeber* help our understanding of human physiological and behavioural rhythmicity (Erren *et al.*, 2020)? Here, we propose that, while so-called clock genes play a clear and firm role in keeping and regulating an organism’s rhythms, a persistent and pervasive oscillatory force such as gravity tide may act as an exogenous driver to many—if not all—internal oscillators of living organisms.

Evidence exists that such a tidal-like synchronism does occur for different cyclic patterns, for example, for wood quality (Zürcher, 2001; Vogt *et al.*, 2002), for the daily variation of tree trunk size (Zürcher *et al.*, 1998; Barlow *et al.*, 2010) and tree stem electrical potential (Barlow, 2012), for root growth (Barlow and Fisahn, 2012; Fisahn *et al.*, 2012; Barlow *et al.*, 2013), for chlorophyll fluorescence (Fisahn *et al.*, 2015), and for the ultra-weak photon emission (UPE) from seedlings (Moraes *et al.*, 2012; Gallep *et al.*, 2017). Here, revisiting existing data from well-documented studies, we also find a tidal synchronism for the swimming activity of isopods kept in free-running conditions (Enright, 1965), and for larvae release in a coral reef, recorded for 6 months after being in controlled, water-tide-free conditions for 16 months (Jokiel *et al.*, 1985). This effect is examined in further detail later on in this review.

A well-known case is that of leaf movements, the rhythmic motion that occurs due to changes in turgor of the pulvini and petioles extensor cells. This kinetic process is regulated by the influx and efflux of water and potassium ions that serve to change cellular volume over time (Sweeney, 1987). Calcium ion content was also recently found to be involved in generating rhythmic leaf motions (Moysset *et al.*, 2019). Such movements, first studied in detail by Darwin (1897), are notably persistent in continuous light (Hoshizaki and Hamner, 1964) and were once considered the ‘Rosetta Stone’ of plant cyclic behaviour (Satter and Galston, 1973), with the related circadian nature of the motion still lacking full understanding (Ueda *et al.*, 2019). Similar results by Brouwer (1926), Kleinhoonte (1929, 1932), Bünning and Stern (1930), and Stoppel (1912, 1916, 1926) on the cyclic motion of either articulated or non-articulated leaves (Schmitz, 1934) promoted the idea of an internal autonomous clock. Most notably, the close inspection and analysis conducted by Barlow (2015) of results available in the literature revealed ubiquitous tidal patterns and synchronism. The first data review of this type contributed by Barlow appeared in 2008, using data from Klein (2007). This meticulous meta-analysis revealed nastic movements of leaf blades that were synchronous with the local gravimetric pattern. Time-resolved analyses of numerous examples indicate that an increasing tidal force usually depresses the leaf downwards, and that rapid leaf bending movements occur when the local *δg* changes its variation, that is, when the gravimetric ‘force changed from either a minimum (“low tide”) or a maximum (“high tide”)’ (Barlow *et al.*, 2008). The extensive data review of Barlow (2015) shows that the oscillatory patterns found in the leaf movements for different species and cultivars are coincident with the patterns of the calculated local gravimetric tide. Barlow (2015) proposes the following important statement:

‘a lunisolar clock, in which the *zeitgeber* is exogenous and independent of metabolism, has been suggested as lying within a category of “primal” phenomena … [that could allow] both animal and plant organisms to continue to express rhythmic patterns of behavior under conditions where light is absent’.

The subsequent independent analysis of more recent data also showed that stem growth, nutation, and leaf movement in young peppermint plants follow the pattern of variation of the local *δg* (Zajączkowska and Barlow, 2017; Zajączkowska *et al.*, 2019), corroborating Barlow’s idea of a tide-related drive for leaf movements. Interestingly, Barlow presents a similar phenomenology from data obtained in the unique conditions of the International Space Station, where a tidal cycle takes just 90min to complete and where leaf movements undergo 90min cycles, coinciding with the local gravimetric patterns of the orbiting space station (Johnsson *et al.*, 2009; Fisahn *et al.*, 2015).

Another example of such cyclic patterns stems from the group of Gallep, who uncovered strong and novel evidence during germination tests, that is, the daily and monthly patterns occurring in the UPE from seedlings exhibiting covariation with lunisolar gravimetric patterns (Gallep *et al.*, 2007). UPE, also known as biological autoluminescence or chemiluminescence, has been reported to occur across many taxa, and is proposed to happen in all living organisms, in a spectrum spanning from near UVA to visible light and near IR (Devaraj *et al.*, 1997). Related to by-products of the metabolic activity, reactive oxygen species and other electronically excited chemical species that occur inside living cells (Cifra and Pospíšil, 2014), UPE can be detected during normal development (Ichimura *et al.*, 1989) or in response to alterations of the organism’s normal physiological state (Makino *et al.*, 1996). Gallep’s group, using UPE data recorded during series of germination tests with different toxic compounds, found that the control groups present UPE patterns that vary from day to day, and also during the course of the month (Gallep *et al.*, 2007; Gallep, 2014). Further series of germination tests were performed, in Gallep’s laboratory and in those of research partners in other countries, working with the contributions of Barlow and colleagues, with the objective of comparing UPE time series with the local *δg* cycles (Moraes *et al.*, 2012; Gallep et al., 2013, 2014, 2017). The collected data—several series of consecutive germination tests from different species—show that seedlings present variations in UPE intensity directly related to sprout growth, and that the UPE time patterns are similar to those of the local *δg*. Further, the UPE for a single sunflower seedling, as one example, also turned out to co-vary with the local *δg* (Gallep 2014), as detailed in the next section.

## Data meta-analysis: interrogation of previous data

In this section, we offer a renewed presentation and interrogation of published data, stemming from studies with temporally and geographically well-documented data and enabling the proper determination of the local gravimetric tide, performed here using the software D-Tide, based on the numerical model of Longmann (1959) [see Appendix at the Repositório de Dados de Pesquisa da Unicamp (REDU), https://doi.org/10.25824/redu/UGMCJV; Gallep and Robert (2021)]. Whenever possible, the intention is to position the data in the time frame of the local gravimetric tide, and to explore temporal relations with it. Hence, we recall here three case studies: the swimming activity of isopods, the release of larvae in a coral reef, and the UPE of single sunflower seedlings.

**Fig. 1.**
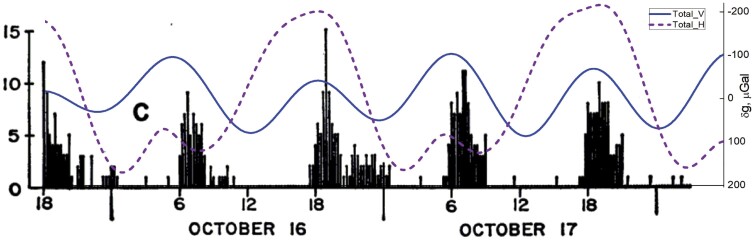
Number of swimming isopods (*Excirolana chiltoni*) in controlled free-running conditions, superposed to the local gravimetric tide (*δg*, μGal): vertical (Total_V, solid blue line) and horizontal (Total_H, dashed purple line) components. Increased swimming appears in conjunction with local *δg* peaks, which in field conditions are the timing of high water tides. From Enright (1965). Entrainment of a tidal rhythm. Science 147, 864–867. Reprinted with permission from AAAS.

The swimming activity of cirolanid isopods, studied by J.T. Enright, constitutes an interesting case. The test animals were collected on 5 October 1964 from the sandy beach in front of the Scripps Institute of Oceanography, La Jolla, CA, USA (32°52ʹ05.3″N 117°15ʹ13.0″W), and then kept in a controlled laboratory (environmental conditions in Enright, 1965). Two groups were entrained by an artificial wave action, and a third group was kept as a control and not swirled about, in a free-running light regime of continuous illumination. The swimming activity of this control group, after having been taken out of the natural environment and kept in ‘free-running’ conditions for 13 d, is reproduced in Fig. 1. To these activity data we have superposed the calculated local gravimetric tide profiles, showing both vertical and horizontal components (see Appendix at https://doi.org/10.25824/redu/UGMCJV). It is noteworthy that the animals were active just after negative peaks of the gravimetric tide, which occur twice a day, in this case at around 06.00h and 18.00 h—a timing that locally related to maxima in the water levels in the natural environment.

Enright’s data on *Excirolana chiltoni* clearly show that rhythmic activity persists in free-running conditions (Fig. 1). An internal circadian clock with ~24h cycles would not coincide with, or track, the onset of swimming activity. After several days in free-running conditions, significant delays would have arisen, and the timing for onset of swimming (the actual best time for swimming activity) would be delayed by about 1h every day. From these data, it becomes apparent that the most robust predictor for the timing of swimming activity is the lunisolar gravimetric tide that dictates the time of arrival of high tide, rather than the light:dark regime dictated by the solar circadian rhythm.

**Fig. 2.**
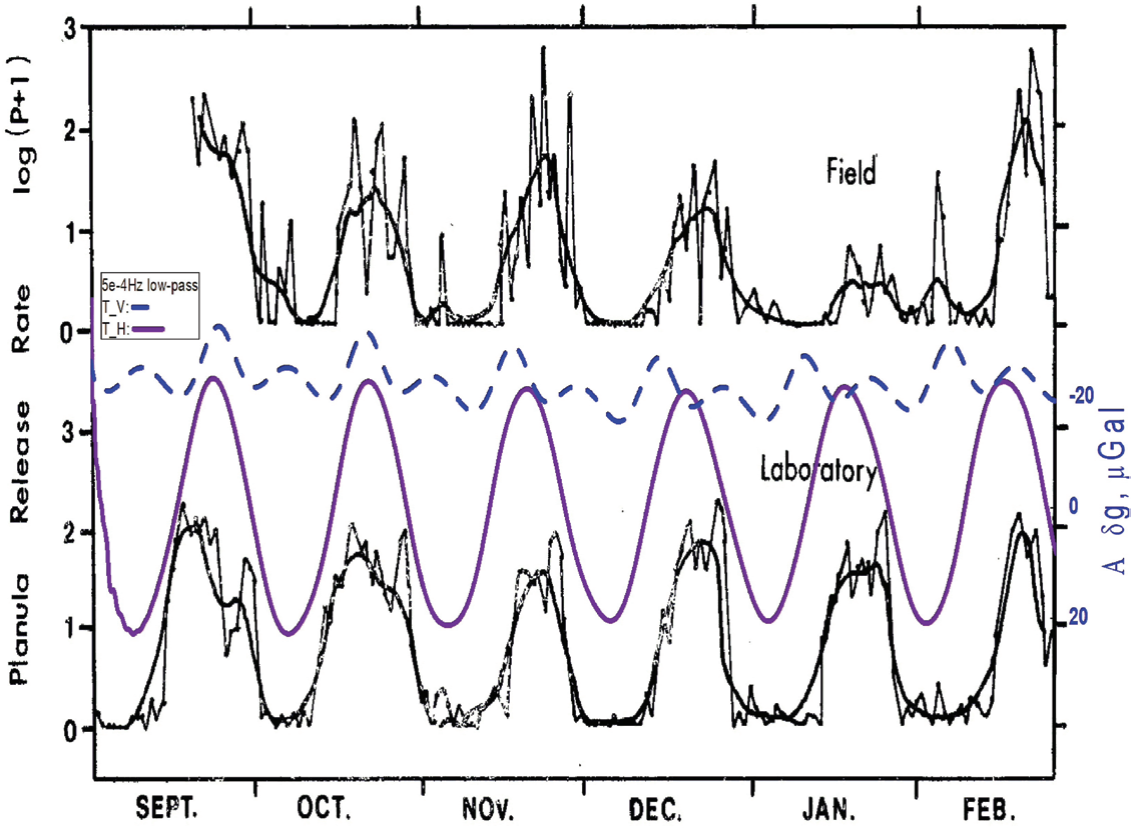
Coral larvae release rate [log(P+1), where P is the number of larvae) (*Pocillopora damicornis*). The jagged curve depicts daily values; the solid smooth line depicts a 5-day average. The amplitude modulation of the local gravimetric tide was calculated, and superposed to the original data by Jokiel *et al.* (1985). Total vertical (T_V, dashed blue line) and total horizontal (T_H, solid purple line) components (low pass-filter for f <5.10^–4^ min^–1^, A_*δg*, μGal). Time scale is in d, starting on 1 September 1980. From Jokiel *et al.*, 1985. Night irradiance and synchronization of lunar release of planula larvae in the reef coral *Pocillopora damicornis*. Marine Biology 88, 167–174. Reprinted by permission from Springer Nature, © 1985.

The release of planula larvae by coral (*Pocillopora damicornis*) in the reef constitutes another well-documented example. Jokiel *et al.* (1985) collected planula larvae at Kaneohe Bay, Hawaii (21°25ʹ59.1″N 157°47ʹ18.7″W) and moved them to laboratories of the nearby Hawaii Institute of Marine Biology, where the organisms were cultivated for 16 months in controlled conditions, with constant water flow, temperature, and salinity. The production of larvae was accurately recorded from September 1980 to February 1981 for field coral and for the population cultivated in the laboratory. The time series of the laboratory planula emergence is reproduced here in Fig. 2, and superposed to the monthly variation in amplitude of the local gravimetric tide, obtained by a low-pass frequency filtering (f <5.10^–4^ min^–1^; see details in Appendix at https://doi.org/10.25824/redu/UGMCJV), showing the long-term changes in the tide excursion occurring due to the lunar cycle of ~28 d. Interestingly, planula emergence in the free-running laboratory situation showed the same periodicity as that of the freshly collected samples from the field. The magnitude of planula emergence peaked at the first quarter and the full moon, that is, at the times of the highest water tides. It is noteworthy that the reproduction cycles of the free-running samples mostly fit the amplitude of the horizontal gravimetric component, which at that time and location is much bigger than the vertical component. In addition, although the Moon phase advances a few days every month and planula release increases a few days later, as seen in data from November onwards, the cessation of planula release is delimited by the tidal cycle, in effect when the tide amplitude decreases towards the end of each month. In Jokiel and colleagues’ study, the authors used artificial Moon irradiance to induce change in rhythmicity in the treated groups. This served to draw the conclusion that Moon light is the cue that maintains the tide-tracking, even for ‘such simple animals lacking complex sensory organs, endocrine systems and neurological systems’ (Jokiel *et al.*, 1985).

**Fig. 3.**
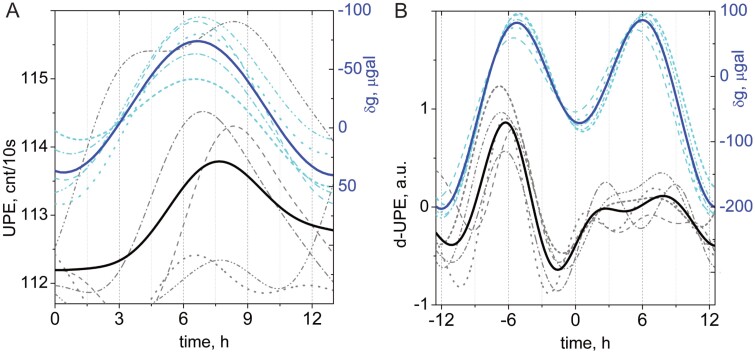
Time patterns of the spontaneous photon emission (UPE, cnt/10s) of sunflower seedlings measured individually (dashed grey lines) and average values (solid black line), and of the local gravimetric tide (*δg*, μGal) at the time and location of the experiments (dashed cyan lines, individual tides; solid blue line, average time course of tide). (A) and (B) show two germination tests conducted in controlled conditions, for 13h and 25h time scales respectively, using UPE data (d-UPE; de-trended from linear growth, for B), as detailed in Gallep (2014). a.u., arbitrary units. See Appendix at https://doi.org/10.25824/redu/UGMCJV for information.

The third revisited case is that of spontaneous UPE measured during the germination of single sunflower seedlings, as reported by Gallep (2014) (Fig. 3). Time-resolved data are displayed as the superposition of the consecutive cycles (see Appendix at https://doi.org/10.25824/redu/UGMCJV) and provide evidence of cycles with oscillation periods around but not exactly on a 12h and 24h basis for both the UPE of seedlings and local gravimetric force. Average data in UPE and gravimetric tide profiles highlight the covariance and delay between tidal force and photon emissions (Fig. 3). Oscillations (Fig. 3A) present evidence for bi-circadian cyclic UPE patterns that sequentially repeat in keeping with the gravimetry tidal curves. The graphs are arranged such that successive gravimetric tides are plotted over each other, keeping the phase of maximal tide coincidental with others. Here, the periods of five cycles show 13h patterns. On a longer time scale spanning a quarter moon cycle of 5 d (Fig. 3B), oscillations of gravimetric tide and UPE follow similar patterns of about but not exactly 12h and 24h that reflect the main components present in the tide patterns. This example highlights the presence of rhythmicity that looks very much like that of a circadian oscillator, yet in the detail of its time course can be linked to the effect of the gravimetric tide.

## Discussion

The proposal that the organization of cyclic patterns in the activity of organisms involves ions and ion-transport channels is nearly 50 years old (Njus *et al.*, 1974). Transmembrane ion transport, foundational to explaining the mechanisms at work in neuronal systems and their signalling, has also been invoked in regulating more general rhythmic activities (Njus *et al.*, 1974; Kohn *et al.*, 2017; Miley *et al*., 2018). It is, however, notable that research on non-transcriptional oscillators lost momentum and slowed down its accumulation of evidence with the discovery in the 1990s of the so-called clock genes, which were identified to orchestrate many rhythmic activities across diverse plant and animal taxa (Takahashi, 1993). Consequentially, alternative models, such as redox cycles working autonomously, were put forth only recently (Milev *et al.*, 2018). Some authors clearly point out recently that, even with ‘clock genes’ being active in the cyclic process, the presence of some external cue—a *Zeitgeber*—such as light, exercise, or food is needed for developing and maintaining the periodic rhythmic regulation of mature cells (du Pré *et al.*, 2014). It is important to express the view here that the timing of information issued by environmental cues has often been shown to be critical for the temporal organization of biological systems, widely expressed as, among many others, patterns of behavioural activity, sleep schedules, variation in body temperature, blood pressure, endocrine cycles, or gene expression (Takahashi, 1993). In effect, the dependence and influence of possible different natural *Zeitgebers* on each other are complex and still poorly understood, while being barely taken into account in laboratory experiments (van der Veen *et al.*, 2017). In view of this, the question of the presence and nature of internal, external, or hybrid clocks, and functional models for them, is far from settled. The simple observation that organisms living at different latitudes are very flexible with their temporal organization, as they track local cycles of light and temperature during the passing seasons of a year, still awaits a proper empirical explanation based on the operation of an internal clock (Hut *et al.*, 2013). The possibility that more than one rhythm, if not many, could run independently in a single cell prompts the notion that a multitude of oscillators, for instance, one for each rhythm, is at work. Albeit not parsimonious, such oscillators are often deemed to connect to each other in a non-linear manner (Roenneberg and Morse, 1993; Lillo *et al.*, 2001). This latter view is remarkably daunting, considering the complexity of coupling non-linear oscillators to the inherently unstable (and potentially genuinely chaotic) nature of outputs, in contrast to the invariably smooth and rather harmonic nature of the observed collective oscillations.

Remarkably, circadian oscillators exist without the involvement of gene transcription, as exemplified by the *in vitro* self-sustainable and cyclic phosphorylation of KaiC cyanobacterial proteins (Nakajima *et al.*, 2005). Endowed with a circadian-like rhythm, phosphorylation was shown to persist even without transcription or translation, and was temperature independent. Quite similarly, heterogeneous reaction networks can generate robust oscillations within complex mixtures comprising precursors that do not oscillate on their own (Wagner and Ashkenasy, 2019). Corroborating the non-necessity of gene transcription for circadian rhythmicity, two studies document, using red blood cells lacking a nucleus, the circadian regulation of glucose metabolism (O’Neill and Reddy, 2011; Ch *et al.*, 2021). In effect, a number of well-documented studies strongly suggest that canonical circadian clock genes (i.e. the homologous clock genes identified in many higher organisms) are not necessary or even may not be involved in the generation of circadian or tidal rhythms and the resulting phenotypes (Bulla *et al.*, 2017).

Current understanding of the mechanisms of gravity sensing in plants rests on a widespread model and rich evidence from numerous studies from the past 100 years (Moulia and Fournier, 2009) based on the sedimentation of dense starch granules inside specialized cells. While evidence for the involvement of starch granules or amyloplasts in the sensing of gravity is overwhelming, sensitivity to gravity may also rely on additional mechanisms and therefore may be more complicated than previously surmised. In effect, plants deprived of such sensing cells can still display gravitropism (Mancuso *et al.*, 2006; Edelmann, 2018). Notably, recent work has shown that amyloplasts are more than passive starch granules, as they undergo constant active mobility, in fact always interacting dynamically with the cytoskeleton (Volkmann *et al.*,1999; Forterre and Pouliquen, 2018). The motion of amyloplasts persists even when they are not exposed to the gravity force (Saito *et al.*, 2005). Remarkably, it was demonstrated recently that plant gravitropism is independent of the magnitude of gravity, with the position of amyloplasts being more important than the force they exert upon the cell wall in determining gravitropic reactions (Chauvet *et al.*, 2016).

While the identity of the actual ‘gravity transducer’ remains unknown, the role of changes in cell membrane fluidity with variations in gravity has been considered (Kohn *et al.*, 2017). Plasma-membrane-based mechanisms, such as mechanosensitive ion channels, have been proposed as a common principle for force sensation (Kung, 2005), because of their exquisite sensitivity to small fluctuations in force, and they are ubiquitous across phyla, from bacteria to mammals (Peyronnet *et al.*, 2014). Such mechanosensitive channels are demonstrably involved with calcium ions in the response of organisms to the variation in micro-gravity (Nazir *et al*., 2014; Bizet *et al.*, 2018). These cell-level mechanisms could also help explain the presence of phonotropism in plants (Rodrigo-Moreno *et al.*, 2017), that is, their reaction to imposed sounds and vibrations (Gagliano *et al.*, 2012).

It has been proposed that the mechanical sensing of small changes in gravity occurs across a large number of cells, beyond the realm of root apical cells containing starch granules. This proposition pertains to the function of large webs of extracellular interlinked microtubules that connect tissue (Nick, 2013). Such an extracellular matrix is thought to mediate long-range mechanical interactions between cells in a tissue (Baluška *et al*., 2004). In this model, individual cells would be disturbed by very weak forces, whereby the gravitational force upon a cell of ~10^–12^ N applied to the cell membrane (10^–8^ m length scale) would lead to an imparted energy of ~10^–20^ J. This quantity of energy is commensurate to that of an action potential relative to the resting membrane potential. The direct effect on the cell might be large enough to elicit variations in membrane potential, alter the probability of channel opening, and/or activate sensitive secondary messaging processes (Persinger, 2014). This proposition is worth considering and testing empirically with modern analytical methods in mechanical biophysics.

It has been pointed out that cellular-level interfaces have complex glass-like interactions with structured aqueous domains, with biophysical properties that are deemed to play a role in the flow of mechanical energy into protein-based mechano-transduction and signalling (Pagnotta, 2005; Hwang, 2018). A better understanding of water clusters inside the cell, and their unusual material properties, would also be necessary to elucidate the proposed roles of water during cyclic forced interactions with a gravimetric tide *Zeitgeber* (Chaplin, 2000; Gadre *et al.*,2014). The role of interfacial and ‘bound’ water in the interactions of biological and mineral material is beginning to be studied and modelled (Tuladhar *et al.*, 2020; Tanaka *et al.*, 2021), shedding light on the modulation of mechanical forces exerted by the gravimetric tide.

In effect, matter does not need to be alive to undergo the effects of the gravimetric tide. Basic physical attributes of matter exhibit periodic oscillations related to the Sun–Moon–Earth cycles, such as the conductivity of water (Ageev, 2018) and the decay of radioactive isotopes (Fischbach *et al.*, 2009; Sturrock *et al.*, 2014). These physical processes are poised to impact on biological processes, such as the electrotonic potentials that are the force driving ion pumps and transmembrane electron and proton transport (de Toledo *et al.*, 2019). More exotically, the possibility has been considered that quantum coherence plays a role in sustaining long-range electron transfer in proteins, a very intriguing form of sequential interaction and isolation of the organism with its environment (Lambert *et al.*, 2013).

Finally, the question arises whether it would be a mere fantasy to propose that the gravimetric tide, an ever-present force acting on every living organism across its evolutionary history, could be a driving force on the internal oscillators of every organism. If, as nicely expressed by Szent-Györgyi 50 years ago, ‘life is water dancing to the tune of the solids’ (Szent-Györgyi, 1971), why would the daily and monthly gravimetric tides not directly act upon organisms, since gravity and its variations act upon all matter?

## Conclusions

We would like to propose, emulating many such prior propositions (i.e. Brown, 1983; Zürcher *et al.*, 1998; Klein, 2007; Persinger, 2014;Barlow, 2015; Zajączkowska *et al.*, 2019), that the interactions of biological organisms with the weak cyclical forces of gravimetric tides constitute a key and determining driver of biological oscillators. We also propose that such forces are sufficient to entrain biological rhythms. Naturally, the enormous role of circadian genes is also recognized here, in view of their cyclic expression and regulation, as part of a tremendously complex signalling network orchestrating physiology and behaviour in a timely manner.

The brief analysis presented here highlights a possible caveat in the phenomenological significance of so-called free-run experiments in the laboratory. Free-running seems to assume that constant light, constant darkness, or constant humidity or temperature imply overall constant conditions and an absence of temporal cues to the organism under test. This is evidently not the case, as several other and distinct physical quantities can vary, and do so with their own cyclic patterns. Such quantities are the gravimetric tide, geomagnetic field fluctuations, daily variations in the atmospheric electric potential gradient, and the flux of cosmic rays. As potential cues, such physical quantities are not easily detectable and controllable in laboratory conditions. Some of them are not even avoidable, such as the tidal variations in gravity presented here. These potential cues have, however, some fundamental temporal characteristics in common, as their cyclic patterns depend on Sun–Moon–Earth orbital dynamics. Thus, an organism’s sensory ecological niche may well offer richer and more structured information than previously surmised. As to the gravimetric tide, it has been acting on Earth for as long as the sun rose and set, and the moon waxed and waned, a discrete but pervasive force that has accompanied the rhythms of life since their beginnings.

## Data Availability

Data on gravimetric tide prediction and validation, ultra-weak photon emissions from sunflower, and gravimetric tide data from Kanehoe and La Jolla (USA) are available at the Repositório de Dados de Pesquisa da Unicamp (REDU) https://doi.org/10.25824/redu/UGMCJV;Gallep and Robert, 2021). Raw data, including the D-Tide software, are available from the corresponding author, CM Gallep, upon request.

## References

[CIT0001] Adushkin VV , Riabova SA, Spivak AA. 2017. Lunar–solar tide effects in the Earth’s crust and atmosphere. Izvestiya, Physics of the Solid Earth 53, 565–580.

[CIT0002] Ageev IM , Rybin YM, Shishkin GG. 2018. Manifestation of solar–terrestrial rhythms in variations of the electrical conductivity of water. Biophysics 63, 282–288.

[CIT0003] Akasofu SI. 1982. Interaction between a magnetized plasma flow and a strongly magnetized celestial body with an ionized atmosphere: energetics of the magnetosphere. Annual Review of Astronomy and Astrophysics 20, 117–138.

[CIT0004] Arnaudon L , Bordry F, Coosemans W, et al. 1993. Effects of tidal forces on the beam energy in LEP. In: International Conference on Particle Accelerators. Washington DC, USA, 17–20 May, 1993. Proceedings. New York: IEEE, 44–46.

[CIT0005] Aschoff J. 1981. Biological rhythms. Boston: Springer.

[CIT0006] Baluska F , Volkmann D, Barlow PW. 2004. Cell bodies in a cage. Nature 428, 371. 15042068 10.1038/428371a

[CIT0007] Barlow PW. 2012. Moon and cosmos: plant growth and plant bioelectricity. In: Volkov AG, ed. Plant Electrophysiology. Berlin: Springer, 249–280.

[CIT0008] Barlow PW. 2015. Leaf movements and their relationship with the lunisolar gravitational force. Annals of Botany 116, 149–187. 26205177 10.1093/aob/mcv096 PMC4512198

[CIT0009] Barlow PW , Fisahn J. 2012. Lunisolar tidal force and the growth of plant roots, and some other of its effects on plant movements. Annals of Botany 110, 301–318. 22437666 10.1093/aob/mcs038 PMC3394636

[CIT0010] Barlow PW , Fisahn J, Yazdanbakhsh N, Moraes TA, Khabarova OV, Gallep CM. 2013. *Arabidopsis thaliana* root elongation growth is sensitive to lunisolar tidal acceleration and may also be weakly correlated with geomagnetic variations. Annals of Botany 111, 859–872. 23532042 10.1093/aob/mct052 PMC3631336

[CIT0011] Barlow PW , Klingelé E, Klein G, Sen MM. 2008. Leaf movements of bean plants and lunar gravity. Plant Signaling & Behavior 3, 1083–1090.

[CIT0012] Barlow PW , Mikulecký M Sr, Střeštík J. 2010. Tree-stem diameter fluctuates with the lunar tides and perhaps with geomagnetic activity. Protoplasma 247, 25–43. 20393759 10.1007/s00709-010-0136-6

[CIT0013] Bizet F , Pereda-Loth V, Chauvet H, Gérard J, Eche B, Girousse C, Courtade M, Perbal G, Legué V. 2018. Both gravistimulation onset and removal trigger an increase of cytoplasmic free calcium in statocytes of roots grown in microgravity. Scientific Reports 8, 11442. 30061667 10.1038/s41598-018-29788-7 PMC6065396

[CIT0014] Boerez J , Hinderer J, Jones MA, Rivera L. 2012. Analysis and filtering of the effect of tides on the hydrostatic levelling systems at CERN. Survey Review 44, 256–264.

[CIT0015] Brouwer G. 1926. De periodieke bewegingen van de primaire bladen bij *Canavalia ensiformis*. Amsterdam: HJ Paris.

[CIT0016] Brown FA Jr . 1954. Persistent activity rhythms in the oyster. American Journal of Physiology 178, 510–514. 10.1152/ajplegacy.1954.178.3.510 13207368

[CIT0017] Brown FA Jr . 1959. Living clocks. Science 130, 1535–1544. 13804924 10.1126/science.130.3388.1535

[CIT0018] Brown FA Jr . 1976. Biological clocks: endogenous cycles synchronized by subtle geophysical rhythms. BioSystems 8, 67–81. 953161 10.1016/0303-2647(76)90010-1

[CIT0019] Brown FA Jr . 1983. The biological clock phenomenon: exogenous timing hypothesis. Biological Rhythm Research 14, 137–162.

[CIT0020] Bruce VG , Pittendrigh CS. 1957. Endogenous rhythms in insects and microorganisms. The American Naturalist 91, 179–195.

[CIT0021] Bulla M , Oudman T, Bijleveld AI, Piersma T, Kyriacou CP. 2017. Marine biorhythms: bridging chronobiology and ecology. Philosophical Transactions of the Royal Society B: Biological Sciences 372, 20160253. 10.1098/rstb.2016.0253 PMC5647280 28993497

[CIT0022] Bünning E , Stern K. 1930. Über die tagesperiodischen Bewegungen der Primärblätter von *Phaseolus multiflorus*. II. Die Bewegungen bei Thermokonstanz. Berichte der Deutschen Botanischen Gesellschaft 48, 227–252.

[CIT0023] Cajochen C , Altanay-Ekici S, Münch M, Frey S, Knoblauch V, Wirz-Justice A. 2013. Evidence that the lunar cycle influences human sleep. Current Biology 23, 1485–1488. 23891110 10.1016/j.cub.2013.06.029

[CIT0024] Ch R , Rey G, Ray S, et al. 2021. Rhythmic glucose metabolism regulates the redox circadian clockwork in human red blood cells. Nature Communications 12, 377. 10.1038/s41467-020-20479-4 PMC7810875 33452240

[CIT0025] Chaplin MF. 2000. A proposal for the structuring of water. Biophysical Chemistry 83, 211–221. 10647851 10.1016/s0301-4622(99)00142-8

[CIT0026] Chauvet H , Pouliquen O, Forterre Y, Legué V, Moulia B. 2016. Inclination not force is sensed by plants during shoot gravitropism. Scientific Reports 6, 35431. 27739470 10.1038/srep35431 PMC5064399

[CIT0027] Cifra M , Pospíšil P. 2014. Ultra-weak photon emission from biological samples: definition, mechanisms, properties, detection and applications. Journal of Photochemistry and Photobiology. B, Biology 139, 2–10. 10.1016/j.jphotobiol.2014.02.009 24726298

[CIT0028] Cresci A , Durif CM, Paris CB, Thompson CRS, Shema S, Skiftesvik AB, Browman HI. 2019. The relationship between the moon cycle and the orientation of glass eels (*Anguilla anguilla*) at sea. Royal Society Open Science 6, 190812. 31824702 10.1098/rsos.190812 PMC6837198

[CIT0029] Darwin C. 1897. The power of movement in plants. New York: D. Appleton and Company.

[CIT0030] de Mairan JJ. 1729. Observation botanique. Paris: Histoire de l’Académie Royale des Sciences.

[CIT0031] de Toledo GR , Parise AG, Simmi FZ, Costa AV, Senko LG, Debono MW, Souza GM. 2019. Plant electrome: the electrical dimension of plant life. Theoretical and Experimental Plant Physiology 31, 21–46.

[CIT0032] Devaraj B , Usa M, Inaba H. 1997. Biophotons: ultraweak light emission from living systems. Current Opinion in Solid State and Materials Science 2, 188–193.

[CIT0033] du Pré BC , van Veen TA, Young ME, Vos MA, Doevendans PA, van Laake LW. 2014. Circadian rhythms in cell maturation. Physiology 29, 72–83. 24382873 10.1152/physiol.00036.2013

[CIT0034] Edelmann HG. 2018. Graviperception in maize plants: is amyloplast sedimentation a red herring? Protoplasma 255, 1877–1881. 29948366 10.1007/s00709-018-1272-7 PMC6208824

[CIT0035] Enright JT. 1965. Entrainment of a tidal rhythm. Science 147, 864–867. 17793561 10.1126/science.147.3660.864

[CIT0036] Erren TC , Schmiedehausen S, Groß JV, Lewis P. 2020. What if …. the Moon provides zeitgeber signals to humans? Molecular Psychiatry 25, 2646–2647. 30116030 10.1038/s41380-018-0216-0

[CIT0037] Fisahn J , Klingelé E, Barlow P. 2015. Lunar gravity affects leaf movement of *Arabidopsis thaliana* in the International Space Station. Planta 241, 1509–1518. 25795423 10.1007/s00425-015-2280-x

[CIT0038] Fisahn J , Yazdanbakhsh N, Klingele E, Barlow P. 2012. *Arabidopsis thaliana* root growth kinetics and lunisolar tidal acceleration. New Phytologist 195, 346–355. 10.1111/j.1469-8137.2012.04162.x 22583121

[CIT0039] Fischbach E , Buncher JB, Gruenwald JT, Jenkins JH, Krause DE, Mattes JJ, Newport JR. 2009. Time-dependent nuclear decay parameters: new evidence for new forces? Space Science Reviews 145, 285–335.

[CIT0040] Forterre Y , Pouliquen O. 2018. Physics of particulate flows: from sand avalanche to active suspensions in plants. Comptes Rendus Physique 19, 271-284.

[CIT0041] Gadre SR , Yeole SD, Sahu N. 2014. Quantum chemical investigations on molecular clusters. Chemical Reviews 114, 12132–12173. 25341561 10.1021/cr4006632

[CIT0042] Gagliano M , Mancuso S, Robert D. 2012. Towards understanding plant bioacoustics. Trends in Plant Science 17, 323–325. 22445066 10.1016/j.tplants.2012.03.002

[CIT0043] Gallep, CM. 2014. Ultraweak, spontaneous photon emission in seedlings: toxicological and chronobiological applications. Luminescence 29, 963–968. 24687546 10.1002/bio.2658

[CIT0044] Gallep CM , Barlow PW, Burgos RC, van Wijk EP. 2017. Simultaneous and intercontinental tests show synchronism between the local gravimetric tide and the ultra-weak photon emission in seedlings of different plant species. Protoplasma 254, 315–325. 26820150 10.1007/s00709-016-0947-1

[CIT0045] Gallep CM , Moraes TA, Cervinková K, Cifra M, Katsumata M, Barlow PW. 2014. Lunisolar tidal synchronism with biophoton emission during intercontinental wheat-seedling germination tests. Plant Signaling & Behavior 9, e28671. 24714075 10.4161/psb.28671 PMC4091565

[CIT0046] Gallep CM , Moraes TA, Dos Santos SR, Barlow PW. 2013. Coincidence of biophoton emission by wheat seedlings during simultaneous, transcontinental germination tests. Protoplasma 250, 793–796. 23011402 10.1007/s00709-012-0447-x

[CIT0047] Gallep CM , Moraes TA, Juliao GO, Santos SR. 2007. Rhythmicities in the spontaneous photon emission of wheat seedlings. In: 2007 SBMO/IEEE MTT-S International Microwave and Optoelectronics Conference. Salvador, Brazil, 29 October–1 November, 2007. Proceedings. New York: IEEE, 713–715.

[CIT0048] Gallep CM , Robert D. 2021. Data from: Are cyclic plant and animal behaviours driven by gravimetric mechanical forces? Repositório de Dados de Pesquisa da Unicamp, V3. 10.25824/redu/UGMCJV PMC8866634 34727177

[CIT0049] Hoshizaki T , Hamner KC. 1964. Circadian leaf movements: persistence in bean plants grown in continuous high-intensity light. Science 144, 1240–1241. 17751495 10.1126/science.144.3623.1240

[CIT0050] Hut RA , Paolucci S, Dor R, Kyriacou CP, Daan S. 2013. Latitudinal clines: an evolutionary view on biological rhythms. Proceedings. Biological Sciences 280, 20130433. 23825204 10.1098/rspb.2013.0433 PMC3712436

[CIT0051] Hwang SG , Hong JK, Sharma A, Pollack GH, Bahng G. 2018. Exclusion zone and heterogeneous water structure at ambient temperature. PLoS One 13, e0195057. 29668733 10.1371/journal.pone.0195057 PMC5905880

[CIT0052] Ichimura T , Hiramatsu M, Hirai N, Hayakawa T. 1989. Two-dimensional imaging of ultra-weak emission from intact soybean roots. Photochemistry and Photobiology 50, 283–286.

[CIT0053] Ievinsh G , Kreicbergs O. 1992. Endogenous rhythmicity of ethylene production in growing intact cereal seedlings. Plant Physiology 100, 1389–1391. 16653134 10.1104/pp.100.3.1389 PMC1075795

[CIT0054] Johnsson A , Solheim BGB, Iversen TH. 2009. Gravity amplifies and microgravity decreases circumnutations in *Arabidopsis thaliana* stems: results from a space experiment. New Phytologist 182, 621–629. 10.1111/j.1469-8137.2009.02777.x 19320838

[CIT0055] Jokiel PL , Ito RY, Liu PM. 1985. Night irradiance and synchronization of lunar release of planula larvae in the reef coral *Pocillopora damicornis*. Marine Biology 88, 167–174.

[CIT0056] Kahn PG , Pompea SM. 1978. Nautiloid growth rhythms and dynamical evolution of the Earth–Moon system. Nature 275, 606–611.

[CIT0057] Klein G. 2007. Farewell to the internal clock: a contribution in the field of chronobiology. New York: Springer Science & Business Media.

[CIT0058] Kleinhoonte A. 1929. Über die durch das Licht regulierten autonomen Bewegungen der Canavalia-Blätter. Archives Néerlandaises des Sciences Exactes et Naturelles Serie B5, 1–110.

[CIT0059] Kleinhoonte A. 1932. Untersuchungen über die autonomen Bewegungen der Primärblätter von *Canavalia ensiformis* DC. Jahrbücher für wissenschaftliche Botanik 75, 679–725.

[CIT0060] Kohn F , Hauslage J, Hanke W. 2017. Membrane fluidity changes, a basic mechanism of interaction of gravity with cells? Microgravity Science and Technology 29, 337–342.

[CIT0061] Kuhlman SJ , Mackey SR, Duffy JF. 2007. Biological rhythms workshop I: introduction to chronobiology. Cold Spring Harbor Symposia on Quantitative Biology 72, 1–6. 18419258 10.1101/sqb.2007.72.059

[CIT0062] Kung C. 2005. A possible unifying principle for mechanosensation. Nature 436, 647–654. 16079835 10.1038/nature03896

[CIT0063] Lambert B , Chen YN, Cheng YC, Li CM, Chen GY, Nori F. 2013. Quantum biology. Nature Physics 9, 10–18.

[CIT0064] Lebert M , Porst M, Hader DP. 1999. Circadian rhythm of gravitaxis in *Euglena gracilis*. Journal of Plant Physiology 155, 344–349. 11542916 10.1016/s0176-1617(99)80115-1

[CIT0065] Leiva FP , Niklitschek EJ, Paschke K, Gebauer P, Urbina MA. 2016. Tide-related biological rhythm in the oxygen consumption rate of ghost shrimp (*Neotrypaea uncinata*). Journal of Experimental Biology 219, 1957–1960. 10.1242/jeb.133785 27099365

[CIT0066] Lillo C , Meyer C, Ruoff P. 2001. The nitrate reductase circadian system. The central clock dogma contra multiple oscillatory feedback loops. Plant Physiology 125, 1554–1557. 11299336 10.1104/pp.125.4.1554 PMC1539380

[CIT0067] Longman IM. 1959. Formulas for computing the tidal accelerations due to the moon and the sun. Journal of Geophysical Research 64, 2351–2355.

[CIT0068] Lüttge U. 2003. Circadian rhythmicity: is the “biological clock” hardware or software? In: Progress in botany, Vol. 64. Berlin, Heidelberg: Springer, 277–319.

[CIT0069] Makino T , Kato K, Iyozumi H, Honzawa H, Tachiiri Y, Hiramatsu M. 1996. Ultraweak luminescence generated by sweet potato and *Fusarium oxysporum* interactions associated with a defense response. Photochemistry and Photobiology 64, 953–956. 8972637 10.1111/j.1751-1097.1996.tb01860.x

[CIT0070] Mancuso S , Barlow PW, Volkmann D, Baluska F. 2006. Actin turnover-mediated gravity response in maize root apices: gravitropism of decapped roots implicates gravisensing outside of the root cap. Plant Signaling & Behavior 1, 52–58. 19521476 10.4161/psb.1.2.2432 PMC2633879

[CIT0071] Mercier A , Sun Z, Baillon S, Hamel JF. 2011. Lunar rhythms in the deep sea: evidence from the reproductive periodicity of several marine invertebrates. Journal of Biological Rhythms 26, 82–86. 21252369 10.1177/0748730410391948

[CIT0072] Milev NB , Rhee SG, Reddy AB. 2018. Cellular timekeeping: it’s redox o’clock. Cold Spring Harbor Perspectives in Biology 10, a027698. 28778867 10.1101/cshperspect.a027698 PMC5932581

[CIT0073] Mironov VL , Kondratev AY, Mironova AV. 2020. Growth of Sphagnum is strongly rhythmic: contribution of the seasonal, circalunar and third components. Physiologia Plantarum 168, 765–776. 31613995 10.1111/ppl.13037

[CIT0074] Moraes TA , Barlow PW, Klingelé E, Gallep CM. 2012. Spontaneous ultra-weak light emissions from wheat seedlings are rhythmic and synchronized with the time profile of the local gravimetric tide. Die Naturwissenschaften 99, 465–472. 22639076 10.1007/s00114-012-0921-5

[CIT0075] Moulia B , Fournier M. 2009. The power and control of gravitropic movements in plants: a biomechanical and systems biology view. Journal of Experimental Botany 60, 461–486. 19264759 10.1093/jxb/ern341

[CIT0076] Moysset L , Llambrich E, Simón E. 2019. Calcium changes in *Robinia pseudoacacia* pulvinar motor cells during nyctinastic closure mediated by phytochromes. Protoplasma 256, 615–629. 30382423 10.1007/s00709-018-1323-0

[CIT0077] Nakajima M , Imai K, Ito H, Nishiwaki T, Murayama Y, Iwasaki H, Tokitaka O, Kondo T. 2005. Reconstitution of circadian oscillation of cyanobacterial KaiC phosphorylation in vitro. Science 308, 414–415. 15831759 10.1126/science.1108451

[CIT0078] Nasir A , Strauch SM, Becker I, et al. 2014. The influence of microgravity on *Euglena gracilis* as studied on Shenzhou 8. Plant Biology 16 Suppl 1, 113–119. 23926886 10.1111/plb.12067

[CIT0079] Nick P. 2013. Microtubules, signalling and abiotic stress. The Plant Journal 75, 309–323. 23311499 10.1111/tpj.12102

[CIT0080] Njus D , Sulzman FM, Hastings JW. 1974. Membrane model for the circadian clock. Nature 248, 116–120. 4818914 10.1038/248116a0

[CIT0081] Nobel Assembly . 2017. The Nobel Prize in Physiology or Medicine 2017—Press release. https://www.nobelprize.org/prizes/medicine/2017/press-release/.Accessed June 2021.

[CIT0082] Oliveira AG , Stevani CV, Waldenmaier HE, Viviani V, Emerson JM, Loros JJ, Dunlap JC. 2015. Circadian control sheds light on fungal bioluminescence. Current Biology 25, 964–968. 25802150 10.1016/j.cub.2015.02.021 PMC4382382

[CIT0083] O’Neill JS , Reddy AB. 2011. Circadian clocks in human red blood cells. Nature 469, 498–503. 21270888 10.1038/nature09702 PMC3040566

[CIT0084] Pagnotta SE , Gargana R, Bruni F, Bocedi A. 2005. Glassy behavior of a percolative water-protein system. Physical Review. E, Statistical, Nonlinear, and Soft Matter Physics 71, 031506. 10.1103/PhysRevE.71.031506 15903434

[CIT0085] Palmer JD. 1973. Tidal rhythms: the clock control of the rhythmic physiology of marine organisms. Biological Reviews 48, 377–418.

[CIT0086] Patke A , Young MW, Axelrod S. 2020. Molecular mechanisms and physiological importance of circadian rhythms. Nature Reviews. Molecular Cell Biology 21, 67–84. 31768006 10.1038/s41580-019-0179-2

[CIT0087] Persinger MA. 2014. Terrestrial and lunar gravitational forces upon the mass of a cell: relevance to cell function. International Letters of Chemistry, Physics and Astronomy 2, 15–21.

[CIT0088] Peyronnet R , Tran D, Girault T, Frachisse JM. 2014. Mechanosensitive channels: feeling tension in a world under pressure. Frontiers in Plant Science 5, 558. 25374575 10.3389/fpls.2014.00558 PMC4204436

[CIT0089] Rodrigo-Moreno A , Bazihizina N, Azzarello E, Masi E, Tran D, Bouteau F, Baluska F, Mancuso S. 2017. Root phonotropism: early signalling events following sound perception in Arabidopsis roots. Plant Science 264, 9–15. 28969806 10.1016/j.plantsci.2017.08.001

[CIT0090] Roenneberg T , Morse D. 1993. Two circadian oscillators in one cell. Nature 362, 362–364. 29634015 10.1038/362362a0

[CIT0091] Saito C , Morita MT, Kato T, Tasaka M. 2005. Amyloplasts and vacuolar membrane dynamics in the living graviperceptive cell of the Arabidopsis inflorescence stem. The Plant Cell 17, 548–558. 15689424 10.1105/tpc.104.026138 PMC548825

[CIT0092] Satter RL , Galston AW. 1973. Leaf movements: Rosetta stone of plant behavior? BioScience 23, 407–416.

[CIT0093] Schmitz H. 1934. Die periodischen Bewegungen der Blättern von *Coleus Penzigii*. Zeitschrift für Botanik 27, 353–411.

[CIT0094] Skov MW , Hartnoll RG, Ruwa RK, Shunula JP, Vannini M, Cannicci S. 2005. Marching to a different drummer: crabs synchronize reproduction to a 14-month lunar-tidal cycle. Ecology 86, 1164–1171.

[CIT0095] Stoppel R. 1912. Über die Bewegungen der Blätter von *Phaseolus* bei Konstanz der Außenbedingungen. Berichte der Deutschen Botanischen Gesellschaft 30, 29–35.

[CIT0096] Stoppel R. 1916. Die Abhängigkeit der Schlafbewegungen von *Phaseolus multiflorus* von vershiedenen Außenfaktoren. Zeitschrift für Botanik 8, 609–684.

[CIT0097] Stoppel R. 1926. Die Schlafbewegungen der Blätter von *Phaseolus multiflorus* in Island zur Zeit der Mitternachtsonne. Planta 2, 342–355.

[CIT0098] Straley SC , Bruce VG. 1979. Stickiness to glass: circadian changes in the cell surface of *Chlamydomonas reinhardi*. Plant Physiology 63, 1175–1181. 16660878 10.1104/pp.63.6.1175 PMC542991

[CIT0099] Sturrock PA , Fischbach E, Jenkins J. 2014. Analysis of beta-decay rates for Ag108, Ba133, Eu152, Eu154, Kr85, Ra226, and Sr90, measured at the Physikalisch-Technische Bundesanstalt from 1990 to 1996. The Astrophysical Journal 794, 42.

[CIT0100] Sweeney BM. 1977. Chronobiology (circadian rhythms). In: The science of photobiology. Boston: Springer, 209–226.

[CIT0101] Sweeney BM. 1987. Rhythmic phenomena in plants. San Diego: Academic Press.

[CIT0102] Szent-Györgyi A. 1971. Biology and pathology of water. Perspectives in Biology and Medicine 14, 239–249. 5546252 10.1353/pbm.1971.0014

[CIT0103] Takahashi JS. 1993. Circadian-clock regulation of gene expression. Current Opinion in Genetics & Development 3, 301–309. 8504256 10.1016/0959-437x(93)90038-q

[CIT0104] Takao M , Shimada T. 2000. Long term variation of the circumference of the SPring–8 storage ring. In: Mitaroff WA, Petit-Jean-Genaz C, Poole J, Meinhard R, Laclare JL, eds. 7th European Particle Accelerator Conference. Vienna, Austria, June 26–30, 2000. Proceedings. Geneva: EPS, 1572–1574.

[CIT0105] Takase T , Ishikawa H, Murakami H, Kikuchi J, Sato-Nara K, Suzuki H. 2011. The circadian clock modulates water dynamics and aquaporin expression in Arabidopsis roots. Plant & Cell Physiology 52, 373–383. 21186174 10.1093/pcp/pcq198

[CIT0106] Takekata H , Numata H, Shiga S, Goto SG. 2014. Silencing the circadian clock gene *Clock* using RNAi reveals dissociation of the circatidal clock from the circadian clock in the mangrove cricket. Journal of Insect Physiology 68, 16–22. 24995838 10.1016/j.jinsphys.2014.06.012

[CIT0107] Tal O , Haim A, Harel O, Gerchman Y. 2011. Melatonin as an antioxidant and its semi-lunar rhythm in green macroalga *Ulva* sp. Journal of Experimental Botany 62, 1903–1910. 21220782 10.1093/jxb/erq378 PMC3060675

[CIT0108] Tanaka M , Morita S, Hayashi T. 2021. Role of interfacial water in determining the interactions of proteins and cells with hydrated materials. Colloids and Surfaces. B, Biointerfaces 198, 111449. 33310639 10.1016/j.colsurfb.2020.111449

[CIT0109] Tran D , Sow M, Camus L, Ciret P, Berge J, Massabuau JC. 2016. In the darkness of the polar night, scallops keep on a steady rhythm. Scientific Reports 6, 32435. 27577847 10.1038/srep32435 PMC5006026

[CIT0110] Tuladhar A , Dewan S, Pezzotti S, Brigiano FS, Creazzo F, Gaigeot MP, Borguet E. 2020. Ions tune interfacial water structure and modulate hydrophobic interactions at silica surfaces. Journal of the American Chemical Society 142, 6991–7000. 32233477 10.1021/jacs.9b13273

[CIT0111] Ueda M , Ishimaru Y, Takeuchi Y, Muraoka Y. 2019. Plant nyctinasty – who will decode the ‘Rosetta Stone’? New Phytologist 223, 107–112. 10.1111/nph.15717 30697767

[CIT0112] van der Veen DR , Riede SJ, Heideman PD, Hau, M, van der Vinne V, Hut RA. 2017. Flexible clock systems: adjusting the temporal programme. Philosophical Transactions of the Royal Society B: Biological Sciences 372, 20160254. 10.1098/rstb.2016.0254 PMC5647281 28993498

[CIT0113] Vogt KA , Beard KH, Hammann S, Palmiotto JO, Vogt DJ, Scatena FN, Hecht BP. 2002. Indigenous knowledge informing management of tropical forests: the link between rhythms in plant secondary chemistry and lunar cycles. AMBIO 31, 485–490. 12436848 10.1579/0044-7447-31.6.485

[CIT0114] Volkmann D , Baluška F, Lichtscheidl I, Driss-Ecole D, Perbal G. 1999. Statoliths motions in gravity-perceiving plant cells: does actomyosin counteract gravity? The FASEB Journal, 13, S143–S147. 10352156 10.1096/fasebj.13.9001.s143

[CIT0115] Wagner N , Ashkenasy G. 2019. Rhythm before life. Nature Chemistry 11, 681–683. 10.1038/s41557-019-0301-2 31341262

[CIT0116] Wehr TA. 2018. Bipolar mood cycles and lunar tidal cycles. Molecular Psychiatry 23, 923–931. 28115741 10.1038/mp.2016.263 PMC5524624

[CIT0117] Wilcockson D , Zhang L. 2008. Circatidal clocks. Current Biology 18, R753–R755. 18786379 10.1016/j.cub.2008.06.041

[CIT0118] Zajączkowska U , Barlow PW. 2017. The effect of lunisolar tidal acceleration on stem elongation growth, nutations and leaf movements in peppermint (*Mentha × piperita* L.). Plant Biology 19, 630–642. 28258604 10.1111/plb.12561

[CIT0119] Zajączkowska U , Kasprzak W, Nałęcz M. 2019. Transitions in nutation trajectory geometry in peppermint (*Mentha × piperita* L.) with respect to lunisolar acceleration. Plant Biology 21, 133–141. 30218478 10.1111/plb.12911

[CIT0120] Zhang L , Hastings MH, Green EW, Tauber E, Sladek M, Webster SG, Kyriacou CP, Wilcockson DC. 2013. Dissociation of circadian and circatidal timekeeping in the marine crustacean *Eurydice pulchra*. Current Biology 23, 1863–1873. 24076244 10.1016/j.cub.2013.08.038 PMC3793863

[CIT0121] Zürcher, E. 2001. Lunar rhythms in forestry traditions – lunar-correlated phenomena in tree biology and wood properties. In: Barbieri C, Rampazzi F, eds. Earth-moon relationships. Dordrecht: Springer, 463–478.

[CIT0122] Zürcher E , Cantiani MG, Sorbetti-Guerri F, Michel D. 1998. Tree stem diameters fluctuate with tide. Nature 392, 665–666.

